# Thrombolytic Therapy During *ex-vivo* Normothermic Machine Perfusion of Human Livers Reduces Peribiliary Vascular Plexus Injury

**DOI:** 10.3389/fsurg.2021.644859

**Published:** 2021-06-17

**Authors:** Omar Haque, Siavash Raigani, Ivy Rosales, Cailah Carroll, Taylor M. Coe, Sofia Baptista, Heidi Yeh, Korkut Uygun, Francis L. Delmonico, James F. Markmann

**Affiliations:** ^1^Department of Surgery, Center for Transplantation Sciences, Massachusetts General Hospital, Boston, MA, United States; ^2^Department of Surgery, Beth Israel Deaconess Medical Center, Boston, MA, United States; ^3^Center for Engineering in Medicine and Surgery, Massachusetts General Hospital, Boston, MA, United States; ^4^Shriners Hospitals for Children, Boston, MA, United States; ^5^Harvard Medical School, Boston, MA, United States; ^6^New England Donor Services (NEDS), Waltham, MA, United States

**Keywords:** liver transplant, tissue plasminogen activator, normothermic machine liver perfusion, peribiliary vascular plexus, biliary injury, split liver technique, mural stroma, donation after circulatory death

## Abstract

**Background:** A major limitation in expanding the use of donation after circulatory death (DCD) livers in transplantation is the increased risk of graft failure secondary to ischemic cholangiopathy. Warm ischemia causes thrombosis and injury to the peribiliary vascular plexus (PVP), which is supplied by branches of the hepatic artery, causing higher rates of biliary complications in DCD allografts.

**Aims/Objectives:** We aimed to recondition discarded DCD livers with tissue plasminogen activator (tPA) while on normothermic machine perfusion (NMP) to improve PVP blood flow and reduce biliary injury.

**Methods:** Five discarded DCD human livers underwent 12 h of NMP. Plasminogen was circulated in the base perfusate prior to initiation of perfusion and 1 mg/kg of tPA was administered through the hepatic artery at T = 0.5 h. Two livers were split prior to perfusion (S1, S2), with tPA administered in one lobe, while the other served as a control. The remaining three whole livers (W1-W3) were compared to seven DCD control liver perfusions (C1-C7) with similar hepatocellular and biliary viability criteria. D-dimer levels were measured at T = 1 h to verify efficacy of tPA. Lactate, total bile production, bile pH, and difference in biliary injury scores before and after perfusion were compared between tPA and non-tPA groups using unpaired, Mann-Whitney tests.

**Results:** Average weight-adjusted D-dimer levels were higher in tPA livers in the split and whole-liver model, verifying drug function. There were no differences in perfusion hepatic artery resistance, portal vein resistance, and arterial lactate between tPA livers and non-tPA livers in both the split and whole-liver model. However, when comparing biliary injury between hepatocellular and biliary non-viable whole livers, tPA livers had significantly lower PVP injury scores (0.67 vs. 2.0) and mural stroma (MS) injury scores (1.3 vs. 2.7).

**Conclusion:** This study demonstrates that administration of tPA into DCD livers during NMP can reduce PVP and MS injury. Further studies are necessary to assess the effect of tPA administration on long term biliary complications.

## Introduction

The liver organ shortage has led to increased utilization of donation after circulatory (DCD) death organs, especially in recent years (1). Nevertheless in 2019, <10% of liver transplants (LTs) came from DCD donors (2). A major limitation in expanding the use of DCD livers in transplantation are unfavorable outcomes secondary to ischemic-type biliary strictures (ITBS) resulting in higher rates of graft failure and retransplantation compared to donation after brain death (DBD) livers (3, 4). Studies report that the incidence of these biliary complications in DCD allografts can be as high as 33–50%. In addition, ITBS are often difficult to treat, responding poorly to endoscopic, and radiologic interventions (5–7).

The prevailing hypothesis for these inferior outcomes is in part related to the warm ischemic time (WIT) incurred by these organs during procurement between the agonal phase and cold *in situ* flush (8). The blood supply to the intrahepatic and extrahepatic biliary system of a transplanted liver allograft is exclusively from branches of the hepatic artery (HA) via the peribiliary vascular plexus (PVP) (9, 10). The obligatory acirculatory phase during WIT results in the stasis of blood and microthrombi formation in the delicate PVP microcirculation, obstructing blood flow to the biliary tree (11). Op den Dries et al. demonstrated that biliary injury results in the loss of deep peribiliary glands (dPBG), which contain the biliary progenitor cells responsible for repopulating the biliary epithelium. Thus, damage and detachment of the epithelial cells of the dPBGs leads to the development of fibrosis and ITBS seen in DCD liver allografts (12). For these reasons, WIT is a known risk factor for ITBS.

A potential option to reduce biliary injury and ITBS incidence is administering tissue plasminogen activator (tPA) into the HA to lyse microthrombi. Several studies have demonstrated this effect clinically, showing that tPA administration prior to liver reperfusion mitigates the risk of developing ITBS in DCD LT (11, 13–16) and reduces intraglomerular microthrombi in renal allografts (17). However, a major concern with this approach in LT is transferring tPA activity from the donor liver to recipients, who are often already experiencing coagulopathy, fibrinolysis, and thrombocytopenia in the early reperfusion period (11). Additionally, enzymes such as tPA are not active at cold temperatures (18), limiting administration of the drug to the critical reperfusion phase.

A solution to gain the clinical benefits of tPA administration in DCD LT without incurring additional bleeding risk for the recipient is *ex vivo* liver normothermic machine perfusion (NMP). *Ex vivo* liver perfusion (EVLP) has rapidly emerged as a platform that can be used to recondition marginal livers prior to transplantation (19–23). Thus, we hypothesized that administration of tPA into the HA of discarded human livers during 12 h of NMP would reduce biliary injury by improving blood flow to the PVP.

## Materials and Methods

### Donor Liver Selection

Human livers that were declined for transplantation from donors with consent for research were obtained through the New England Donor Services (NEDS) and LiveOnNy Organ Procurement Organizations. Inclusion criteria included DCD livers, age ≥21, WIT <2 h, cold ischemic time (CIT) <12 h, and no cirrhotic or fibrotic morphology. Livers that were hepatitis B positive, hepatitis C positive, or human immunodeficiency virus (HIV) positive were also excluded. Table 1 details the donor characteristics of discarded liver grafts undergoing NMP. This study was approved by NEDS, LiveOnNY, and exempt by the Massachusetts General Hospital institutional review board (IRB number 2011P001496) as not meeting the definition of human subjects research. No organs were procured from prisoners, and no vulnerable populations were included in this study.

**Table 1:** Detailed characteristics of discarded liver grafts undergoing normothermic machine perfusion (NMP).

**Liver number**	**S1**	**S2**	**W1**	**W2**	**W3**	**C1**	**C2**	**C3**	**C4**	**C5**	**C6**	**C7**	***p*-value**
DCD	Y	Y	Y	Y	Y	Y	Y	Y	Y	Y	Y	Y	
tWIT (min)	38	21	26	29	28	34	26	28	21	24	24	23	0.48
fWIT (min)	18	10	11	13	9	18	9	7	7	9	8	11	0.65
CIT (min)	301	250	750	720	490	557	784	557	210	358	300	357	0.17
Age (years)	59	46	51	61	58	60	21	40	59	46	58	55	0.37
Gender	M	M	F	M	M	F	M	M	M	M	M	F	
BMI (kg/m^2^)	26.6	33.3	26.8	22.1	31.4	32.9	27.4	24.0	29.9	40.2	28.3	24.6	0.46
Weight (kg)	72.5	95.0	73.0	78.0	104	74.1	84.0	76.0	90.9	100	87.0	65.0	0.78
ABO	A	O	A	O	O	O	O	O	A	O	A	O	
Ethnicity	W	W	W	W	W	W	W	W	W	W	W	W	
Smoker	Y	N	N	N	N	Y	N	N	Y	N	Y	N	
Alcohol	1	2	0	0	2	1	1	1	0	0	0	0	
Drug use	0	2	0	0	0	0	0	1	0	0	2	0	
AST (U/L)	39	108	63	40	172	59	82	42	-	594	41	24	0.73
ALT (U/L)	21	146	51	26	79	40	97	58	-	474	22	11	0.56
Tbili (mg/dl)	0.3	0.3	0.4	1	0.8	0.3	0.4	0.3	-	0.5	0.9	0.2	0.16
ALP (U/L)	37	89	273	85	90	-	234	106	-	99	36	94	0.59

*DCD, donation after circulatory death; tWIT, total warm ischemic time; fWIT, functional warm ischemic time; CIT, cold ischemic time; BMI, body mass index (kg/m2), Y, yes. N, no. tPA, tissue plasminogen activator. S, splits. W, whole livers with tPA. C, whole control livers (no tPA). NA, not applicable. Gender: M, male; F, female*.

*ABO blood type: A, B, O*.

*Ethnicity: W, White (non-Hispanic); A, African-American*.

*Smoker: N, non-smoker; Y, active smoker*.

*EtOH: 0, none; 1, social/low use; 2, heavy use (10+ drinks/day)*.

*Drug use: 0, none; 1, former use; 2, active use (includes marijuana)*.

*AST (aspartate aminotransferase), ALT (alanine aminotransferase), Tbili (total bilirubin), and ALP (alkaline phosphatase) levels indicate last available values prior to procurement. “-“ indicates data not available. p-values compare whole tPA livers (W1-W3) to control livers with no tPA (C1-C7), Significance level p ≤ 0.05*.

### Donor Liver Procurement

Each liver was procured in standard fashion utilizing University of Wisconsin (UW) solution for cold flush and preservation. Total WIT (tWIT) was defined as the time from withdrawal of life support to cold *in situ* flush, and functional WIT (fWIT) was defined as the time from circulatory arrest to cold *in situ* flush.

### Split-Liver Technique

The split-liver perfusion model is described in detail in (24). Briefly, an anatomic spilt of the liver into right and left lobes was performed on the back table. The portal vein (PV), hepatic artery (HA), and common bile duct (CBD) were dissected to the level of their bifurcations. The gallbladder was removed and the cystic duct and artery was ligated. The liver parenchyma was divided along Cantlie's line. The hepatic arteries of each lobe were cannulated with 10-12F cannulas and the portal veins with 24F cannulas (Organ Assist, Groningen, The Netherlands). Each bile duct was cannulated with a 4 mm acorn tip cannula (Medtronic, Minneapolis, MN, USA). The hepatic veins were left uncannulated. The cut surfaces of the liver lobes were sealed with Loctite medical grade adhesive (Henkel Corp., Rocky Hill, CT, USA) to prevent excess bile leakage. The lobes were weighed immediately before and after NMP and functional data was normalized to preperfusion weight (24). Finally, a pre-pump flush consisting of lactated ringers (LR), 0.05 mg/ml of solumedrol (Pfizer, New York City, NY USA), and 1 ug/L of Epoprostenol (GlaxoSmithKline, Research Triangle Park, NC, USA) was flushed with 1 L through the PV and 0.5 L through the HA for each split. Two split liver perfusions (labeled S1 and S2) were conducted, with tPA administered into alternative lobes with each perfusion. Comparisons were made between the tPA and control lobes.

### Whole-Liver Technique

In the whole-liver perfusion model, the HA, PV, and CBD were dissected free and cannulated on the back table. Briefly, the gallbladder was removed and the cystic duct and artery was ligated. The HA was cannulated with a 12F cannula, and the PV with a 24F cannula (Organ Assist, Groningen, The Netherlands). The CBD was cannulated with a 4 mm acorn tip cannula (Medtronic, Minneapolis, MN, USA). The hepatic veins were left uncannulated. The liver was weighed immediately before and after NMP and functional data was normalized to preperfusion weight. Finally, a pre-pump flush consisting of lactated ringers (LR), 0.05 mg/ml of solumedrol (Pfizer, NYC, NY, USA), and 1 ug/L of Epoprostenol (GlaxoSmithKline, Research Triangle Park, NC, USA) was flushed with 2L through the PV and 1L through the HA. Three whole livers with tPA (labeled W1-W3) were compared to seven whole livers with no-tPA (labeled C1-C7).

### Normothermic Machine Perfusion (NMP)

Split and whole livers were perfused using the Liver Assist device (Organ Assist, Groningen, The Netherlands). The perfusate consisted of 1L of packed red blood cells (pRBCs), 600 ml of LR, 300 ml of 25% albumin (Grifols Therapeutics, Research Triangle Park, NC, USA), 20,000 units of heparin (Pfizer, New York City, NY, USA) and 8.4% sodium bicarbonate (Hospira, Lake Forest, Il, USA) as needed for a pH of 7.35–7.45. The temperature of the Liver Assist was initially set at 20°C and increased gradually to 37°C within the first 30 min of perfusion. The perfusate was oxygenated with a gas flow rate of 450–700 ml/min (95% O_2_ mix). The initial pressures of the HA and PV were 30 and 4 mmHg, respectively. Both pressures were raised steadily with the HA range of 70–100 mmHg and PV range of 6–10 mmHg with a titration of PV flow:HA flow of 2:1 by 30 min of perfusion. Throughout the perfusion, Clinimix E (TPN) (Baxter Healthcare, Deerfield, Il, USA) supplemented with 30 units of insulin and 25 grams of 50% dextrose in water (D50W) was infused at 30 ml/h, 0.02 g/ml of taurocholic acid (Sigma-Aldrich, St. Louis, MO, USA) was infused at 3 ml/h, and Epoprostenol infused at 8 ug/h. HA and PV resistance were defined as pressure divided by weight-adjusted flow rate. Hemodynamic perfusion parameters and cumulative bile production were recorded at regular time intervals for the full 12 h of NMP. Our NMP technique is described in detail in (25).

### Plasminogen and tPA Administration

Ten ug/ml of human plasminogen (BioVision, San Francisco, CA, USA) was circulated in the base perfusate prior to initiation of perfusion and 1 mg/kg (based on liver weight) of tPA (Alteplase) (Genentech, San Francisco, CA, USA) was delivered though the HA at T = 0.5 h once the liver was warm. Non-tPA livers did not have plasminogen or tPA added to the base perfusate.

### Biochemical Profiling

Perfusate samples were taken every 30 min for the first 4 h and then every 3 h until completion of perfusion to assess bile composition and blood gas analysis. These metrics (pH, BUN, glucose, and lactate) were analyzed using an i-STAT blood Analyzer (Abbott Point of Care Inc., Princeton, NJ, USA). AST and ALT were measured using a Piccolo Hepatic Function Panel (Abaxis, inc., Union City, CA, USA). Efficacy of tPA function in the liver was assessed by D-dimer levels [a fibrin degradation product that is increased in fibrinolysis (26)] at T = 1 h, 30 min after tPA administration. This allowed D-dimer levels to be measured when the tPA (half-life 5 min) had reached steady state in the perfusion system. D-dimer perfusate (outflow) levels were measured with a Human D-Dimer ELISA Kit (ab260076) (Abcam, Cambridge, MA, USA).

Livers were assessed for both NMP hepatocellular viability and biliary viability criteria for transplantation established by de Vries et al. (27). Hepatocellular viability criteria were (1) arterial lactate remaining <1.7 mmol/L within 4 h of NMP and (2) total bile production >10 ml by 4 h of NMP. Of note, both hepatocellular viability criteria had to be met for a liver to be deemed viable for transplantation. The biliary viability criteria was bile pH >7.45 within 4 h of NMP (27). All tPA livers failed to meet hepatocellular or biliary viability criteria, and as a result, were compared to DCD, non-viable liver perfusions from our control biobank (C1-C7).

### Histological Evaluation

Full circumferential, axial, extrahepatic bile duct biopsies were taken before and after perfusion, fixed in formalin, embedded in paraffin, cut, and stained with hematoxylin and eosin (H&E). A blinded pathologist (IR) evaluated the CBD biopsies (specifically the biliary epithelium, mural stroma, peribiliary vascular plexus, thrombosis formation, intramural bleeding, periluminal peribiliary glands (pPBG), deep peribiliary glands (dPBG), and for the presence of inflammation) based on the bile duct injury scoring system from Hansen et al. (28) (Supplementary Table 1) before and after perfusion. Ki67 staining (SAB4501880, Sigma-Aldrich, St. Louis, MO, USA) was also conducted for extra-hepatic bile duct samples as a marker of cellular proliferation.

### Statistical Analysis

Data was analyzed using Prism 8 software (GraphPad Inc., La Jolla, CA, USA). Perfusion metrics were reported as means with standard deviations (SDs) as error bars. D-dimer levels and bile duct injury (BDI) scores were compared between tPA and non-TPA groups with two-tailed, unpaired Mann-Whitney (non-parametric) tests. To ensure valid comparisons, values were normalized to lobar or whole-liver weight. A *p* ≤ 0.05 was considered statistically significant.

## Results

### Donor Characteristics

This study included 12 discarded DCD human livers: 2 underwent split-liver NMP (with one lobe receiving tPA and the other lobe serving as the control), and the remaining 10 livers underwent whole-liver NMP. The mean donor age was 52 ± 11 years, the mean tWIT was 26 ± 4.9 min, the mean fWIT was 11 ± 3.6 min, and the mean CIT was 469 ± 194 min in the cohort. There were no significant differences in whole-liver donor characteristics between the tPA (W1-W3) and non-tPA (C1-C7) groups with respect to tWIT, fWIT, CIT, age of donor, body mass index (BMI), or liver function tests (Table 1).

### Perfusion Parameters

**Figure 1:**
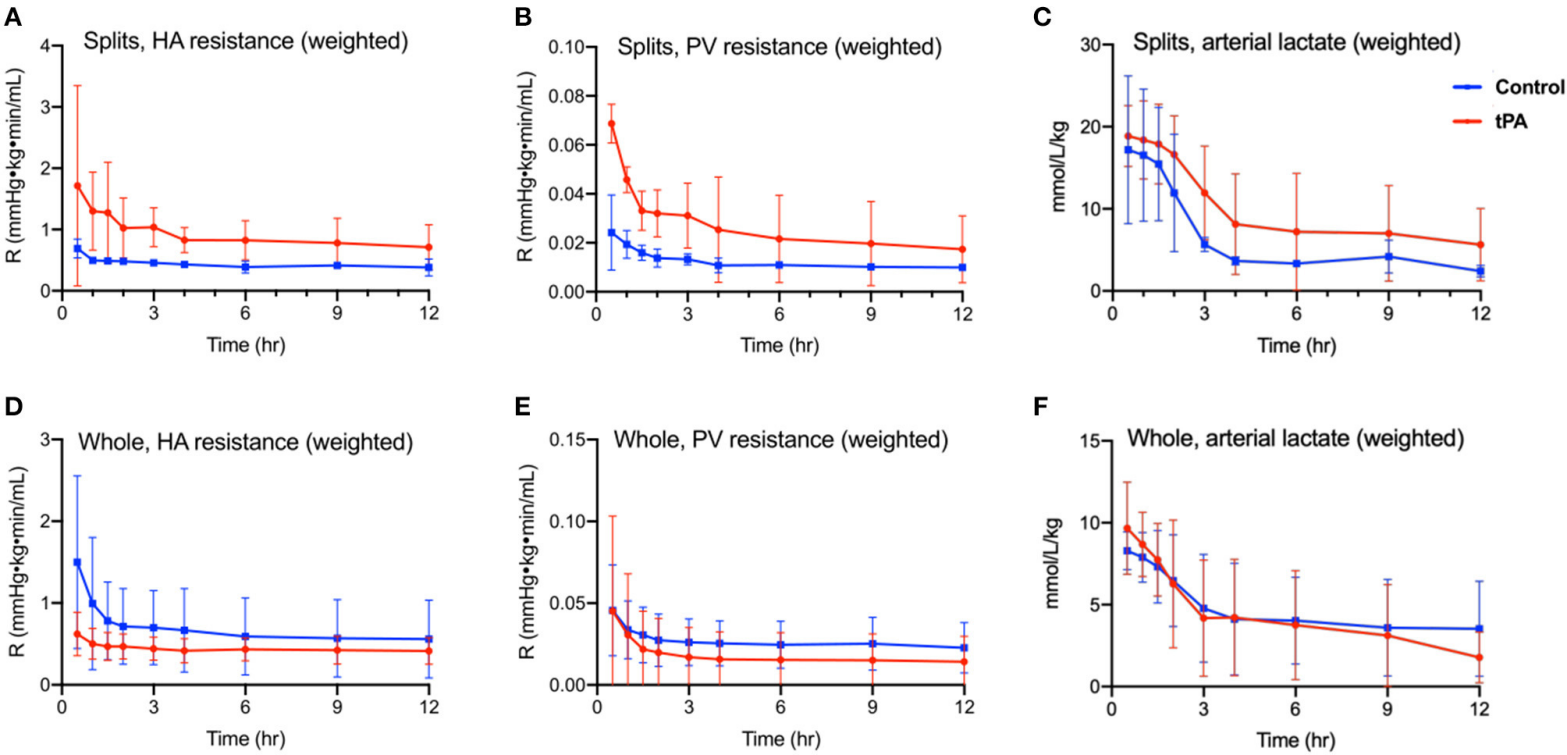
Perfusion metrics of 12 h normothermic machine perfusion (NMP). Split liver model comparing **(A)** hepatic artery resistance (defined as hepatic artery pressure divided by hepatic artery flow rate, adjusted for liver weight), **(B)** portal vein resistance (defined as portal vein pressure divided by portal vein flow rate, adjusted for liver weight), and **(C)** arterial (inflow) lactate (adjusted for liver weight) showed no statistically significant differences between tPA lobes (red) and control lobes (blue) on two-way ANOVA testing. Whole liver model comparing **(D)** hepatic artery resistance, **(E)** portal vein resistance, and **(F)** arterial (inflow) lactate (adjusted for liver weight) showed no statistically significant differences between tPA whole livers (red) and control livers (blue) on two-way ANOVA testing. HA, hepatic artery. PV, portal vein. R, intrahepatic resistance. Error bars indicate standard deviations. Significance level *p* ≤ 0.05.

Weight-adjusted HA resistance (Figure 1A) and PV resistance (Figure 1B) decreased in both split liver lobes over the 12 h of NMP with no significant differences between tPA lobes and non-TPA lobes after liver stabilization on pump. Analogously, arterial lactate decreased steadily in both split liver lobes, with no significant differences between tPA lobes and non-TPA lobes at any timepoint (Figure 1C). Similar to the split-liver model, whole livers also demonstrated slight decreases in HA resistance (Figure 1D), stable PV resistance (Figure 1E), and steadily decreasing arterial lactate levels (Figure 1F), with no significant differences between tPA livers and non-tPA livers at any timepoint. There were also no significant differences in AST, ALT, arterial pH, perfusate BUN, and perfusate glucose levels between tPA and non-tPA split lobes (Supplementary Figure 1) and whole livers (Supplementary Figure 2).

### Viability Assessment

**Figure 2:**
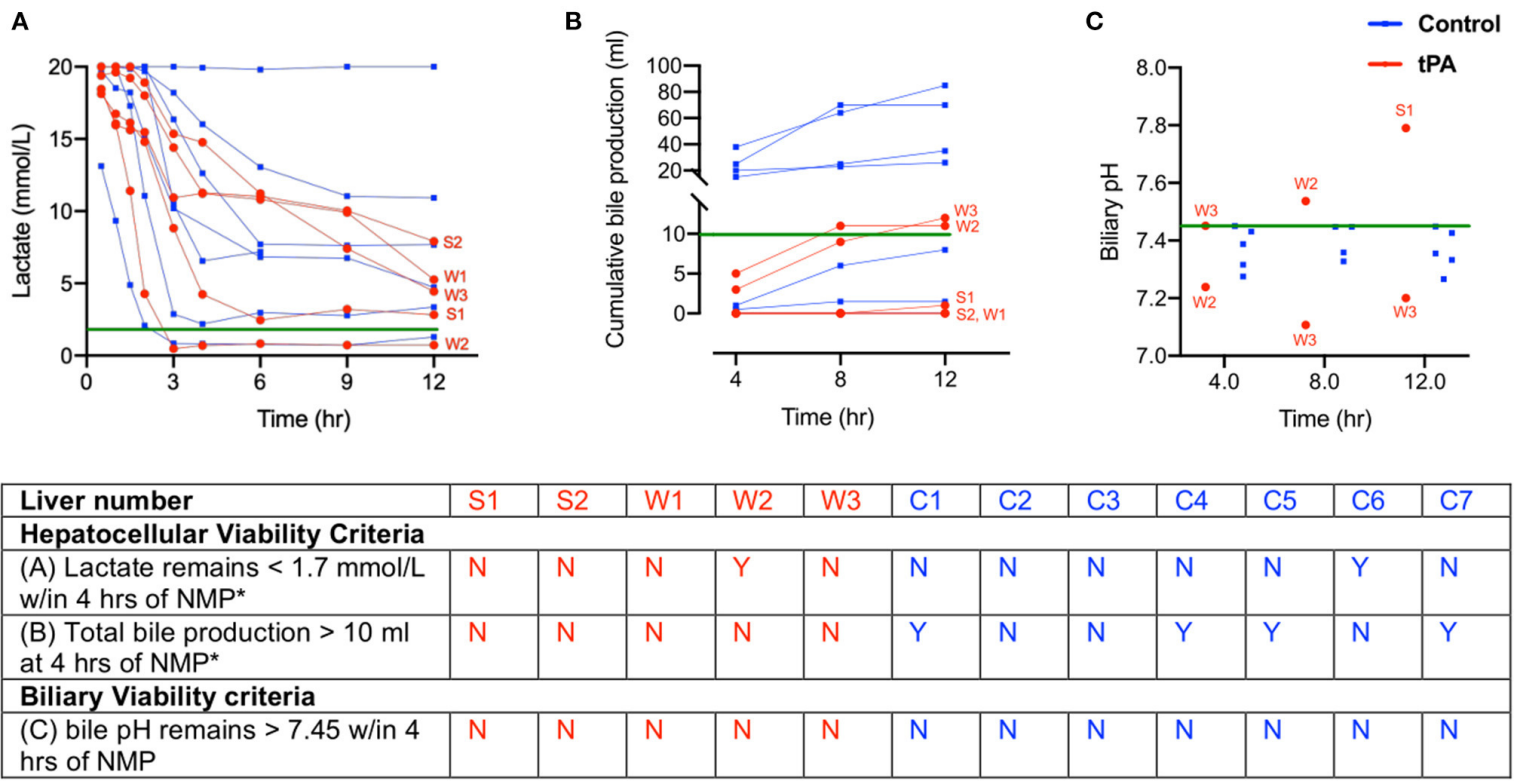
Donor liver assessment during normothermic machine perfusion (NMP) shows all livers are non-viable with both hepatocellular and biliary criteria. **(A)** Inflow lactate levels **(B)** cumulative bile production and **(C)** bile pH in tPA livers (red) and control livers (blue). Green lines indicate viability criteria. Table summarizes hepatocellular and biliary viability criteria for transplantation. *Both hepatocellular viability criteria must hold true for a liver to be viable. tPA, tissue plasminogen activator. W, whole livers with tPA. S, split liver lobe with tPA. C, whole liver controls without tPA. bPH, biliary pH. Y, yes. N, no.

All liver perfusions were assessed for NMP hepatocellular and biliary viability criteria for transplantation (27). Regarding arterial lactate, only livers W2 and C6 had lactate levels <1.7 mmol/L within 4 h of NMP. The remaining livers never reached the 1.7 mmol/L threshold, and C2 did not clear lactate at all (Figure 2A). Next, none of the tPA livers produced 10 ml of bile within 4 h of NMP, making them all non-viable since both criteria needed to be met for hepatocellular viability. Control livers C1, C4, C5, and C7 were able to meet the bile production threshold, but did not meet the lactate clearance criteria, making all control livers (C1-C7) non-viable from a hepatocellular standpoint (Figure 2B). Finally, none of the livers included in this study met the biliary viability criteria of bile pH remaining >7.45 within 4 h of NMP. S1 had a bile pH of 7.79, but only after 12 h of NMP, and bile pH values for W2 and W3 did not remain >7.45 throughout the entire perfusion (Figure 2C). Thus, the comparisons between tPA and non-tPA livers in this study were all made between livers that did not meet hepatocellular or biliary transplant viability criteria.

### Efficacy of tPA

**Figure 3:**
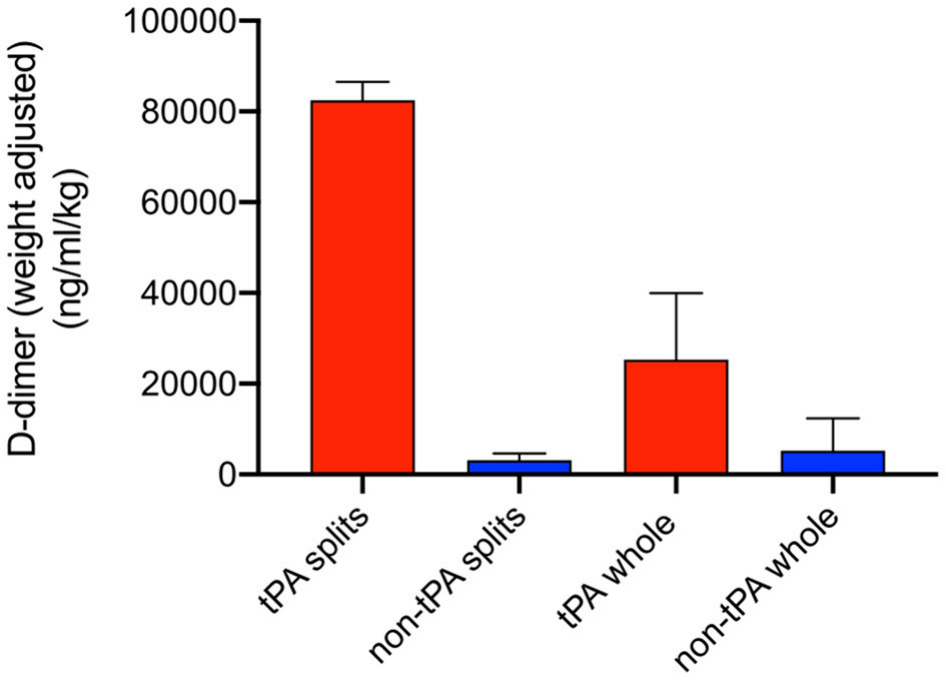
Weight-adjusted perfusate D-dimer levels at T = 1 h of normothermic machine perfusion (NMP). Higher D-dimer levels were seen in tPA splits and tPA whole livers compared to non-tPA controls. Error bars represent standard deviation.

D-dimer perfusate levels were measured at 1 h of NMP, 30 min after administration of tPA, to confirm thrombolytic activity within the perfusion system. Levels were adjusted for liver weight. Split liver lobes with tPA (right S1, left S2) had higher D-dimer levels compared to their control lobes (left S1, right S2) (82,459 vs. 3,122 ng/ml/kg). Whole tPA livers (W1-W3) also had significantly higher D-dimer levels compared to non-tPA controls (C1-C7) (25,296 vs. 5,224 ng/dl/kg, *p* = 0.03) (Figure 3).

### Bile Duct Injury (BDI) and Ki67 Analysis

BDI was assessed using the scoring system by Hansen et al. (28) and Op den Dries et al. (12) (Supplementary Table 1). Overall, split liver lobes had similar BDI scores between tPA and non-tPA lobes. Both splits did not have any PVP injury before and after perfusion (BDI score of 0). Both sets of splits (right S1 & left S1, and right S2 & left S2) had the same mural stroma (MS) BDI score after perfusion of grade 1 and grade 2, respectively. Both sets of splits also had the same periluminal peribiliary gland BDI scores (0 for S1 and grade 2 for S2) after perfusion. The tPA split lobes did have more intramural bleeding (IMB) after perfusion compared to the control lobes. Full BDI scoring for split liver lobes is reported in Supplementary Table 2. Based on the similarities of these BDI results between the split-liver groups, we decided to test the remaining livers with a whole-liver model.

**Figure 4:**
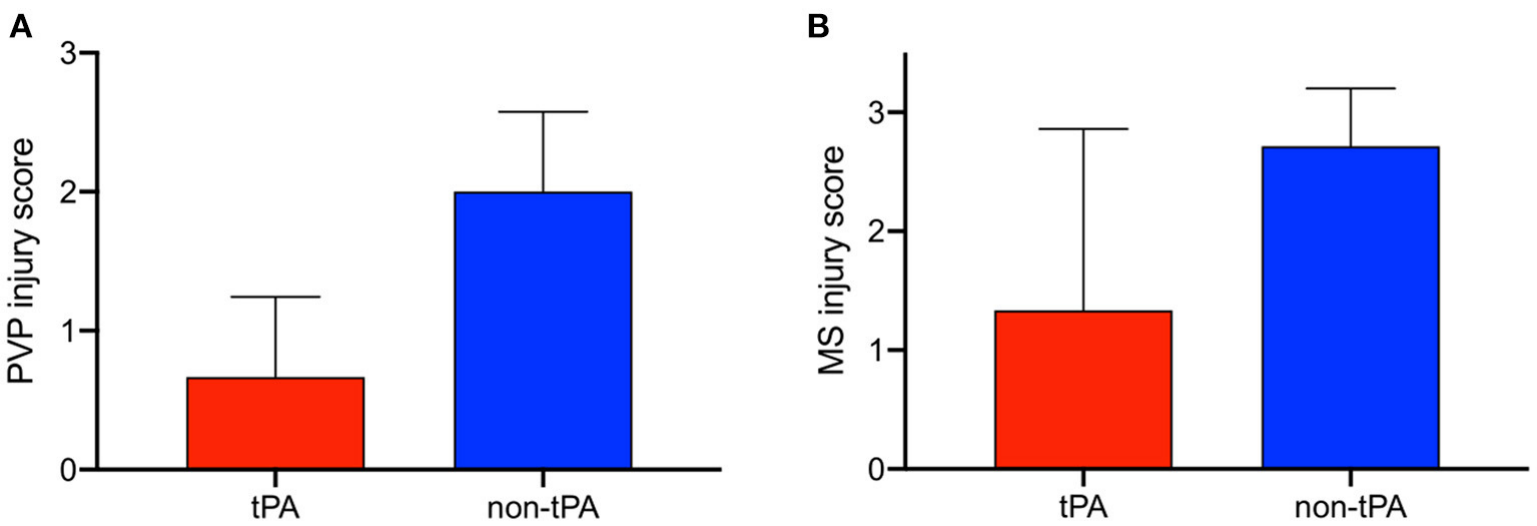
Difference in bile duct injury scores before and after 12 h of normothermic machine perfusion (NMP) between whole, non-viable tPA livers (red) and whole, non-viable control livers (blue) for peribiliary vascular plexus (PVP) injury and mural stroma (MS) injury. tPA livers exhibit less **(A)** PVP injury and **(B)** MS injury. Error bars represent standard deviation.

**Figure 5:**
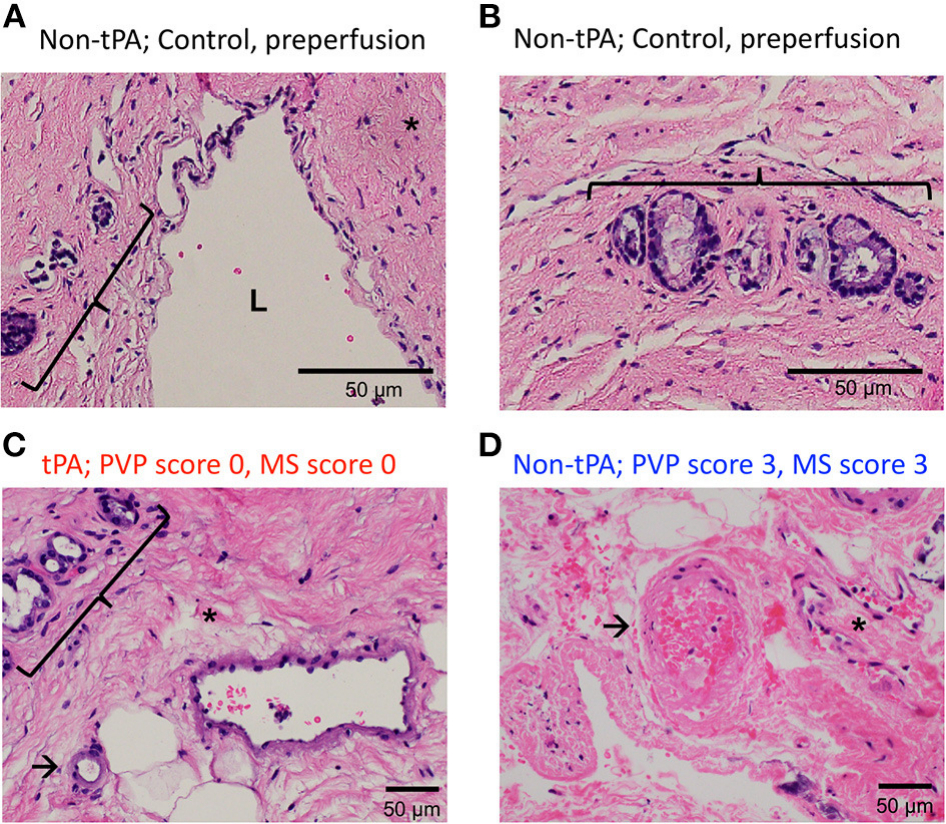
Representative H&E histology of preperfusion common bile ducts **(A)** show bile duct lumen (L) and normal mural stroma (asterisk), periluminal peribiliary glands (bracket) and **(B)** deep peribiliary glands (bracket). H&E after 12 h of NMP of tPA liver **(C)** shows normal peribiliary arterioles (arrows), unremarkable peribiliary glands (bracket), and no injury to MS (asterisk) vs. non-tPA liver histology **(D)** shows arteriolonecrosis (arrow) and necrotic mural stroma (asterisk) indicative of grade 3 injury.

In contrast to the split lobes, there were significant differences between whole tPA livers (W1-W3) and whole controls (C1-C7) with respect to PVP and MS injury. The average change in PVP BDI score before and after perfusion for tPA livers was 0.67 vs. 2 for control livers (*p* = 0.025). Similarly, the average change in MS BDI score before and after perfusion for tPA livers was 1.33 vs. 2.71 for control livers. Thus, whole-tPA livers (W1-W3) had less PVP and MS injury compared to whole controls (C1-C7) (Figure 4). Additionally, there was virtually no intramural bleeding (IMB) in the entire whole-liver cohort. Full BDI scoring of whole livers is reported in Supplementary Table 3. Representative H&E histology showed non-tPA whole livers before perfusion with normal biliary epithelium (BE), mural stroma, periluminal peribiliary glands (pPBG) (Figure 5A), and deep peribiliary glands (dPBG) (Figure 5B). TPA livers were able to retain intact peribiliary arterioles and PBGs after perfusion (Figure 5C) while non-tPA livers had significant arteriolonecrosis of the PVP indicative of grade 3 BDI and necrotic mural stroma (Figure 5D).

Finally, Ki67 staining was utilized to assess cellular proliferation after 12 h of NMP in the extra-hepatic bile ducts of tPA livers. Supplementary Figure 3 shows the confirmed proliferation of biliary epithelial cells within the peribiliary glands.

## Discussion

One of the major issues in DCD LT is the high incidence of ITBS secondary to the obligatory period of WIT, leading to microthrombi in the PVP which can cause biliary ischemia (13). Interventions using thrombolytic therapy during reperfusion have shown to reduce ITBS, but harbor significant concerns for bleeding (13). In this study, we demonstrate that the administration of tPA into DCD livers during NMP can reduce biliary injury to the PVP without significant intramural bleeding.

The efficacy and safety of recombinant tPA has been extensively studied in the setting of ischemic strokes (29–31) but its assimilation into the field of DCD LT is relatively new. As a result, there is a large variation in tPA protocols with respect to dose, timing (intervention at procurement, intervention at reperfusion, or both), and type of thrombolytic flush. Proponents of intervention at procurement argue that more effective thrombolysis can be achieved (14) while advocates for intervention at reperfusion correctly state that thrombolytics work optimally in normothermia (32).

Postreperfusion bleeding is another major concern with intra-operative administration of tPA. Hashimoto et al. defined postreperfusion bleeding as intraoperative bleeding due to fibrinolysis or coagulopathy that required more than 2 h to obtain adequate hemostasis after portal reperfusion. In their study, 14 out of 22 patients (64%) with tPA administration into the donor HA on the backable had excessive postreperfusion bleeding. The authors acknowledge other factors in addition to tPA administration can contribute to excessive bleeding such as poor graft quality and prior laparotomy, but the study did not have a control group for comparison (11). A reason for such high rates of bleeding with tPA administration during reperfusion is that during the anhepatic phase of a LT, there is reduced coagulation factor synthesis. Additionally, fibrinolytic activity in the LT recipient increases due to a lack of tPA clearance (33). Even post-reperfusion, patients can experience accelerated release of tPA from the new liver allograft endothelium, leading to hyperfibrinolysis (34). Given this intrinsic coagulopathy of a LT recipient during and after reperfusion, there is concern that even small doses of iatrogenic tPA administration during this time can lead to significant hemorrhage (13). EVLP provides a platform to assess the effect of tPA dosing and timing on biliary injury without incurring any additional bleeding risk for patients.

In our study, the administration of 1 mg/kg of tPA (based on liver weight) into split liver lobes and whole livers on pump showed no effect on perfusion hemodynamics, lactate, bile production, or bile composition. However, significant differences in whole-liver PVP BDI scores between tPA and non-tPA livers suggest that a major location of action of thrombolysis in the perfusion system was in the distal branches of the HA supplying the PVP, lysing obstructive microthrombi. The half-life of tPA is only 5–10 min in humans (35), which explains the significant differences in D-dimer perfusate levels at T = 1 h in both the split and whole-liver models. As seen in studies on ischemic stroke, the short half-life of tPA also opens up possibilities for higher dose tPA boluses given over a few minutes while a DCD liver is on EVLP without significantly risking intrahepatic bleeding (35).

Additionally, we demonstrated that tPA reduces PVP injury, which is known to supply the PBGs where biliary progenitor cells are located (36, 37). Newly formed biliary epithelial cells migrate to the luminal surface of bile ducts via small canals in the PBGs to restore the epithelial lining of the bile duct lumen (38, 39). However, in our study, there were no differences in PBG BDI scores (defined as loss of cells, Supplementary Table 1) between tPA and non-tPA livers. A potential explanation is that while there is injury to the PBGs incurred in all DCD liver transplants due to the mandatory WIT (12), 12 h of normothermic perfusion is not enough time for the biliary progenitor cells to proliferate and repopulate the PBGs, which would reduce BDI score. This is also why no livers had improved dPBG or pPBG scores after 12 h of perfusion. Future clinical studies that involve administering tPA to DCD livers on NMP and subsequently transplanting them into recipients can help elucidate whether the improved PVP blood flow from tPA has long-term clinical benefits on PBG biliary progenitor cell repopulation. In addition, the use of regenerative markers such as proliferating cell nuclear antigen (PCNA) and Ki67 can be utilized after *ex-vivo* NMP and transplantation to assess if tPA plays a role in the regeneration of the biliary epithelium.

While the efficacy of tPA in the NMP system and its effect on mitigating whole-liver PVP and MS injury were apparent, the split-liver BDI score results were ambiguous. There are a few potential reasons for this finding. First, splitting the liver takes ~2 h, compared to 30 min for back table preparation of whole livers, which prolongs CIT for the split liver lobes and is damaging to the biliary tree. Second, not all human livers are initially suitable for split due to anatomic reasons such as multiple, small arterial branches or variant arterial anatomy. This situation requires hepatic artery reconstruction that can alter perfusion to the biliary tree once the liver lobe is on pump. Finally, there is a significant amount of dissection required to isolate the lobar bile ducts which can devascularize them from the onset of perfusion (24). Damage to the bile duct during dissection can also make tPA lobes more susceptible to intramural bleeding, which was seen in our splits. Collectively, these factors can confound split-liver BDI data and make post-perfusion control and treatment BDI scores similar. However, at this time, whole-liver BDI results are more clinically relevant, since DCD liver grafts are unlikely to be split for clinical purposes. Additionally, we demonstrated D-dimer levels were higher in tPA splits compared to control lobes, affirming the efficacy of the split-liver *ex situ* machine perfusion system as a platform to test therapeutic interventions.

Another limitation in this study was that all tPA livers were non-viable, and thus compared to non-viable controls. The administration of tPA resulted in reduced PVP injury but did not seem to rescue non-viable livers to make them clinically usable. Potential reasons for the poor biliary viability in tPA livers were the severely injured nature of discarded DCD grafts upon arrival, long CITs in transport of research organs to our lab, and a relatively short perfusion time course for tPA to recondition the bile ducts compared to clinical transplantation. However, if tPA administration is implemented in an appropriate clinical setting in DCD LT with minimal CIT and NMP reconditioning following procurement, the effects of tPA in improving PVP blood flow might be substantial enough to convert biliary non-viable livers into viable organs for transplant.

Despite these limitations, this is the first study to our knowledge to demonstrate that tPA administration into the HA of DCD livers during NMP can reduce PVP injury, which can offset the systemic effects of tPA in LT recipients. As EVLP becomes increasingly adopted into clinical practice, future studies are necessary to determine the optimal weight-based dosing of tPA without causing intrahepatic and intramural bleeding on pump. The implications of establishing universal guidelines for tPA administration for DCD liver grafts on NMP are tremendous as they have the potential to greatly expand the utilization of DCD liver allografts by reducing the incidence of ischemic biliary complications.

## Data Availability Statement

The original contributions presented in the study are included in the article/Supplementary Material, further inquiries can be directed to the corresponding author/s.

## Author Contributions

OH wrote the manuscript. OH, SR, CC, and TC performed the liver perfusions. IR analyzed the histology. OH, SB, and SR performed the D-dimer ELISA assays. OH and SR preformed the statistical analysis. OH, SR, HY, KU, and JM designed the experiment. All authors participated in the critical revision of the manuscript for intellectual content.

## Conflict of Interest

KU is an inventor on multiple patents relevant to this study, has a financial interest in Organ Solutions, a company focused on developing organ preservation technology, and his interests are managed by the MGH and Partners HealthCare in accordance with their conflict of interest policies. The remaining authors declare that the research was conducted in the absence of any commercial or financial relationships that could be construed as a potential conflict of interest.

## Abbreviations

BE: biliary epithelium
BDI: bile duct injury
CBD: common bile duct
CIT: cold ischemic time
dPBG: deep peribiliary glands
DCD: donation after circulatory death
EVLP: *ex-vivo* liver perfusion
HA: hepatic artery
H&E: hematoxylin and eosin
IMB: intramural bleeding
ITBS: ischemic-type biliary strictures
LT: liver transplant
MS: mural stroma
NEDS: New England Donor Services
NMP: normothermic machine perfusion
pPBG: periluminal peribiliary glands
PV: portal vein
PVP: peribiliary vascular plexus
tPA: tissue plasminogen activator
tWIT: total warm ischemic time
fWIT: functional warm ischemic time
UW: University of Wisconsin
WIT: warm ischemic time.
