# Supplementary material for: Thrombolytic Therapy During ex-vivo Normothermic Machine Perfusion of Human Livers Reduces Peribiliary Vascular Plexus Injury
Source: Front Surg. 2021 Jun 17;8:644859. doi: 10.3389/fsurg.2021.644859 (PMC8245781; doi:10.3389/fsurg.2021.644859)
Supplement: Supplementary file 1 [file Table_1.docx]

**Supplemental Table 1**: Bile duct injury scoring system used in assessment of degree of injury.

| Bile duct wall component | Grade 0 | Grade 1 | Grade 2 | Grade 3 |
| --- | --- | --- | --- | --- |
| Biliary epithelium | No loss | ≤ 50% loss | > 50% loss | n.a. |
| Mural stroma | No injury | ≤ 25% necrotic | 25-50% necrotic | > 50% necrotic |
| Peribiliary vascular plexus | No injury | ≤ 50% of vessels with changes | > 50% of vessels with changes | Grade 2 + arteriolonecrosis |
| Thrombosis | Absent | Present | n.a. | n.a. |
| Intramural bleeding | None | < 50% of duct wall | > 50% of duct wall | n.a. |
| Periluminal PBG | No injury | < 50% loss of cells | > 50% loss of cells | n.a. |
| Deep PBG | No injury | < 50% loss of cells | > 50% loss of cells | n.a. |
| Inflammation | None | At least 10 leukocytes / HPF | At least 50 leukocytes / HPF | n.a. |

^* Modified from Hansen et al. (28)^

^PBG, peribiliary gland. HPF, high powered field.^

^n.a., not applicable^
