# Supplementary material for: Thrombolytic Therapy During ex-vivo Normothermic Machine Perfusion of Human Livers Reduces Peribiliary Vascular Plexus Injury
Source: Front Surg. 2021 Jun 17;8:644859. doi: 10.3389/fsurg.2021.644859 (PMC8245781; doi:10.3389/fsurg.2021.644859)
Supplement: Supplementary file 2 [file Table_2.docx]

**Supplemental Table 2**: Split liver bile duct injury scores.

| Liver # |  | BE | MS | PVP | Thm | IMB | pPBG | dPBG | Inf |
| --- | --- | --- | --- | --- | --- | --- | --- | --- | --- |
| Split 1 | Pre | 1 | 0 | 0 | 0 | 0 | 0 | 0 | 0 |
|  | S1R 12h (tPA) | nv | 1 | 0 | 0 | 2 | 0 | 1 | 0 |
|  | S1L 12h | nv | 1 | 0 | 0 | 0 | 0 | 0 | 0 |
| Split 2 | Pre | 1 | 0 | 0 | 0 | 0 | 0 | 0 | 0 |
|  | S2R 12h | 2 | 2 | 0 | 0 | 0 | 2 | nv | 0 |
|  | S2L 12h (tPA) | 2 | 2 | 0 | 0 | 1 | 2 | nv | 0 |

^S, split liver. R, right lobe. L, left lobe. tPA, tissue plasminogen activator (red). control (blue).^

^Pre, prior to perfusion. 12h, after 12 hours of normothermic machine perfusion.^

^BE, biliary epithelium. MS, mural stroma. PVP, peribiliary vascular plexus. Thm, thrombosis. IMB, intramural bleeding. pPBG, periluminal peribiliary gland. dPBG, deep peribiliary gland. Inf, inflammation.^

^nv, not visualized^

^Scores 0-3. 0, no injury. 3, most significant injury. Scoring system from Hansen et al. (28), supplemental table 1.^
