# Supplementary material for: Thrombolytic Therapy During ex-vivo Normothermic Machine Perfusion of Human Livers Reduces Peribiliary Vascular Plexus Injury
Source: Front Surg. 2021 Jun 17;8:644859. doi: 10.3389/fsurg.2021.644859 (PMC8245781; doi:10.3389/fsurg.2021.644859)
Supplement: Supplementary file 3 [file Table_3.docx]

**Supplemental Table 3**: Whole liver distal common bile duct injury scores.

| Liver # |  | BE | MS | PVP | Thm | IMB | pPBG | dPBG | Inf |
| --- | --- | --- | --- | --- | --- | --- | --- | --- | --- |
| W1 | Pre | 1 | 0 | 0 | 0 | 0 | 0 | 0 | 0 |
|  | 12h | 2 | 3 | 1 | 1 | 1 | 2 | 2 | 2 |
| W2 | Pre | 2 | 1 | 1 | 0 | 0 | 1 | 1 | 0 |
|  | 12h | 2 | 2 | 2 | 1 | 0 | 2 | 2 | 0 |
| W3 | Pre | 1 | 0 | 0 | 0 | 0 | 0 | 1 | 0 |
|  | 12h | 2 | 0 | 0 | 0 | 0 | 1 | 1 | 0 |
| C1 | Pre | 2 | 0 | 1 | 0 | 0 | 2 | nv | 0 |
|  | 12h | 2 | 3 | 2 | 1 | 0 | 2 | 2 | 0 |
| C2 | Pre | 1 | 0 | 0 | 0 | 0 | 0 | 0 | 0 |
|  | 12h | 2 | 3 | 2 | 1 | 0 | 2 | 2 | 0 |
| C3 | Pre | nv | 0 | 0 | 0 | 0 | nv | 0 | 0 |
|  | 12h | 2 | 3 | 2 | 0 | 0 | 2 | 1 | 0 |
| C4 | Pre | 2 | 0 | 0 | 0 | 0 | nv | nv | 0 |
|  | 12h | 2 | 3 | 2 | 0 | 0 | 2 | 2 | 0 |
| C5 | Pre | 2 | 1 | 0 | 0 | 0 | nv | nv | 0 |
|  | 12h | 2 | 3 | 3 | 0 | 1 | 2 | nv | 0 |
| C6 | Pre | 2 | 0 | 0 | 0 | 0 | 1 | 0 | 0 |
|  | 12h | 2 | 2 | 2 | 0 | 0 | 2 | 1 | 0 |
| C7 | Pre | 2 | 0 | 0 | 0 | 0 | 2 | 1 | 0 |
|  | 12h | 2 | 3 | 2 | 0 | 0 | 2 | 2 | 0 |

^W, whole livers with tPA (red). C, whole control livers without tPA (blue)^

^Pre, prior to perfusion. 12h, after 12 hours of normothermic machine perfusion.^

^BE, biliary epithelium. MS, mural stroma. PVP, peribiliary vascular plexus. Thm, thrombosis. IMB, intramural bleeding. pPBG, periluminal peribiliary gland. dPBG, deep peribiliary gland. Inf, inflammation.^

^nv, not visualized^

^Scores 0-3. 0, no injury. 3, most significant injury. Scoring system from Hansen et al. (28), supplemental table 1.^
