# Supplementary figures and images for: Thrombolytic Therapy During ex-vivo Normothermic Machine Perfusion of Human Livers Reduces Peribiliary Vascular Plexus Injury
Source: Front Surg. 2021 Jun 17;8:644859. doi: 10.3389/fsurg.2021.644859 (PMC8245781; doi:10.3389/fsurg.2021.644859)

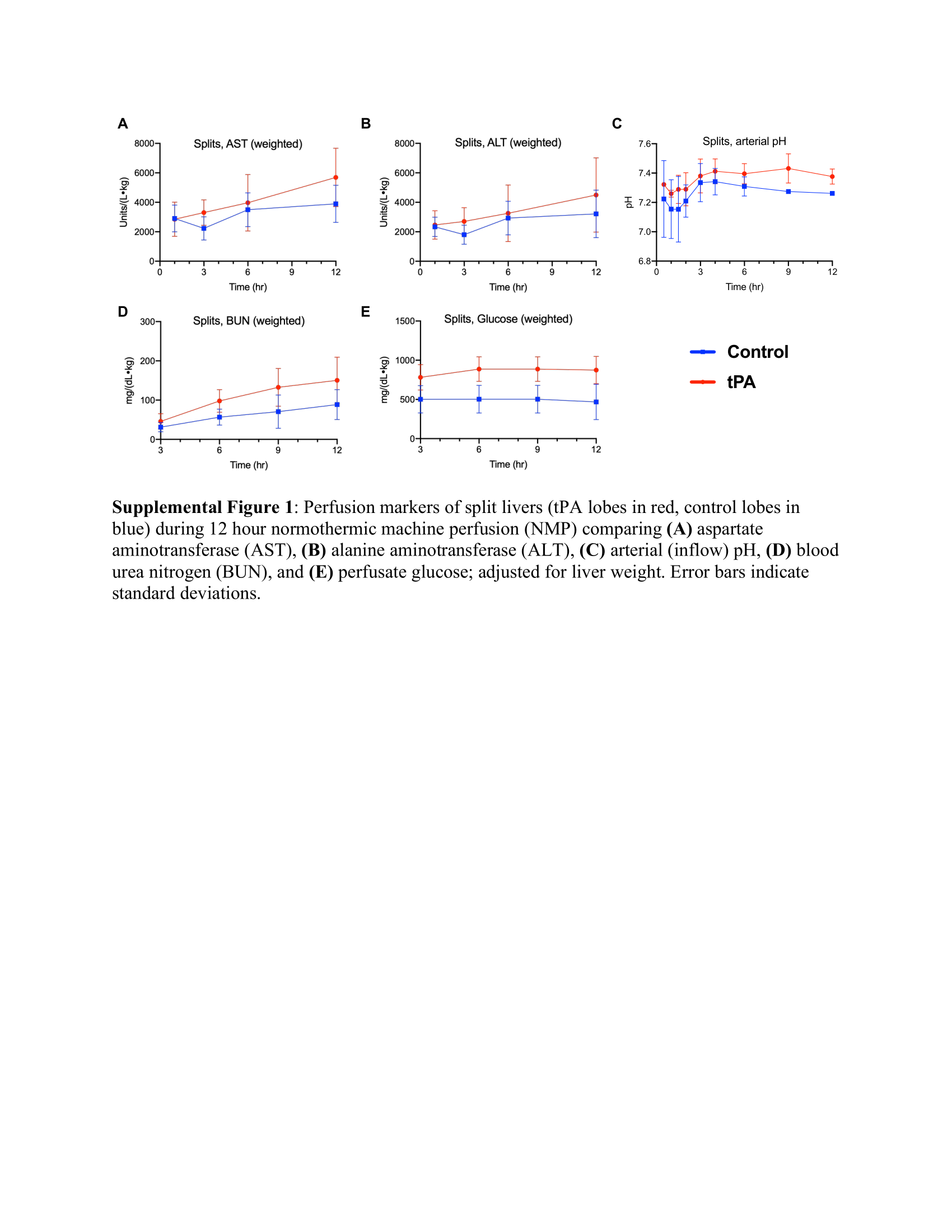

Supplement: Supplementary file 4 [file Image_1.tiff]

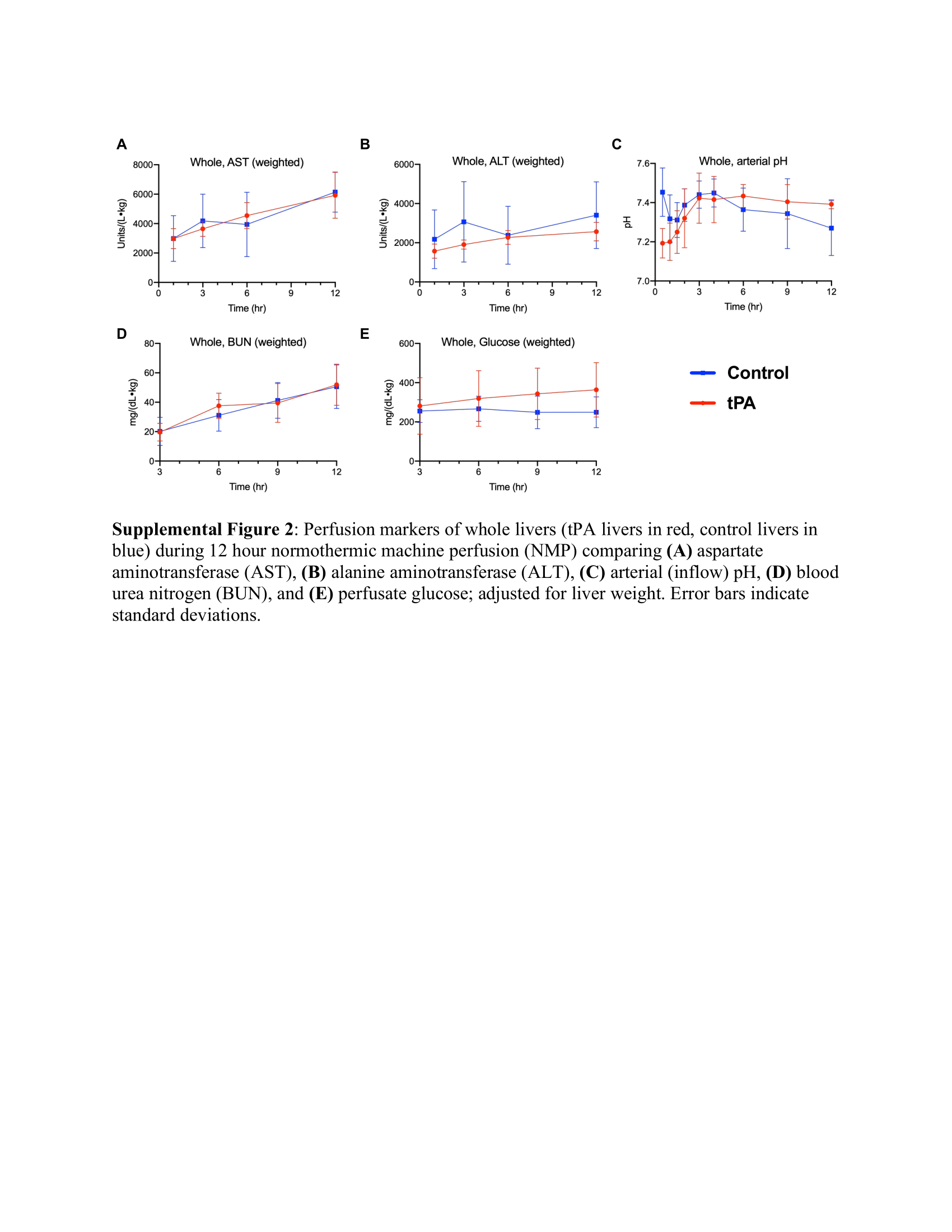

Supplement: Supplementary file 5 [file Image_2.tiff]

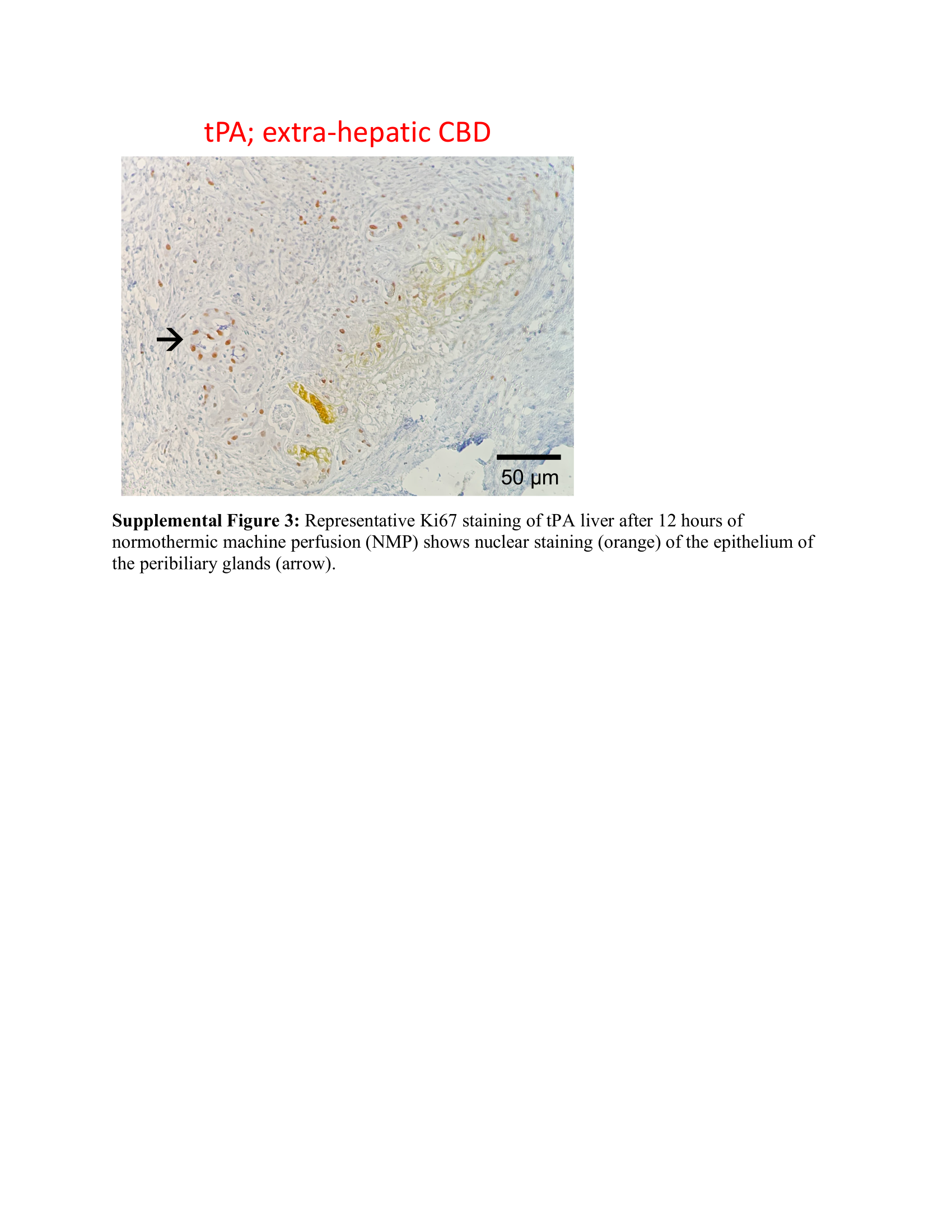

Supplement: Supplementary file 6 [file Image_3.tiff]
